# Clinical Characteristics of Aldosterone- and Cortisol-Coproducing Adrenal Adenoma in Primary Aldosteronism

**DOI:** 10.1155/2018/4920841

**Published:** 2018-03-25

**Authors:** Lu Tang, Xintao Li, Baojun Wang, Xin Ma, Hongzhao Li, Yu Gao, Liangyou Gu, Wenyuan Nie, Xu Zhang

**Affiliations:** ^1^State Key Laboratory of Kidney Disease, Department of Urology, Chinese PLA Medical Academy, Chinese People's Liberation Army General Hospital, Beijing, China; ^2^Department of Urology, Air Force General Hospital, Beijing, China

## Abstract

Aldosterone- and cortisol-coproducing adrenal adenoma (A/CPA) cases have been observed in patients with primary aldosteronism (PA). This study investigated the incidence, clinical characteristics, and molecular biological features of patients with A/CPAs. We retrospectively identified 22 A/CPA patients from 555 PA patients who visited the Chinese People's Liberation Army General Hospital between 2004 and 2015. Analysis of clinical parameters revealed that patients with A/CPAs had larger tumors than those with pure APAs (*P* < 0.05). Moreover, they had higher proportions of cardiovascular complications, glucose intolerance/diabetes, and osteopenia/osteoporosis compared to the pure APA patients (*P* < 0.001). In the molecular biological findings, quantitative real-time PCR analysis revealed similar CYP11B1 and CYP17A1 mRNA expressions in resected A/CPA specimens and in pure APA specimens. Western blot and immunochemical analyses showed CYP11B1, CYP11B2, and CYP17A1 expressions in both A/CPAs and pure APAs. Seventeen cases with KCNJ5 mutations were detected among the 22 A/CPA DNA samples, but no PRKACA or other causative mutations were observed. Each patient improved following adrenalectomy. In conclusion, A/CPAs were not rare among PA patients. These patients associated with high incidences of cardiovascular events and metabolic disorders. Screening for excess cortisol secretion is necessary for PA patients.

## 1. Introduction

Primary aldosteronism (PA) as an endocrine hypertension syndrome was first described by Conn in 1955 [1]. PA is the most common form of secondary hypertension, accounting for more than 10% of patients with hypertension. PA occurs due to excess aldosterone production, which results in moderate to severe hypertension, hypokalemic alkalosis, low plasma renin activity, water-sodium retention, and other disorders [2, 3]. PA can be caused by idiopathic unilateral or bilateral adrenal hyperplasia, aldosterone-producing adenomas (APAs), pure aldosterone-producing adrenocortical carcinomas, familial hyperaldosteronism, and, in rare cases, by ovarian tumors. PA patients with severe or therapy-resistant hypertension have higher risks of cardiovascular damage than patients with the same degree of essential hypertension, independent of age and sex [4–6]. Patients with hypercortisolism also present with hypertension, hypokalemic alkalosis, and an increased risk of cardiometabolic complications, including morbid obesity, impaired glucose tolerance, type 2 diabetes, vertebral osteoporosis, hypercoagulability, and cardiovascular abnormalities [7]. A number of cases of hypercortisolism have been reported in PA patients without the unique clinical features of overt Cushing's syndrome (CS) [8–10]. Subtle excess cortisol secretion may add to the adverse effects of aldosteronism and enhance cardiovascular risks [7]. A previous study reported that PA patients with autonomous cortisol secretion had a high incidence of cardiovascular events, which may increase the risk of cardiovascular damage [11]. Aldosterone- and cortisol-coproducing adenomas (A/CPAs) may have different clinicopathological and biochemical features than pure APAs; if A/CPA can be identified at the initial diagnosis, potential misinterpretation of laboratory test results and possible postoperative adrenal insufficiency could be avoided. In addition, more active surveillance and preventive measures against A/CPA may reduce the risk of cardiovascular damage.

Since the first case was described in 1979, the number of reported cases of A/CPA tumors has been increasing [12]. However, the incidence of aldosterone- and cortisol-cosecreting adrenal tumors remains unclear, with mostly case reports found in the literature [13–16]. Accordingly, aldosterone- and cortisol-cosecreting adenomas may be underrecognized, which may result in mistakes in clinical practice and in crises after tumor resection. Thus, more detailed information about the clinical presentation, hormonal parameters, perioperative preparation, and molecular biological characteristics of A/CPA patients are needed.

With this in mind, we conducted this retrospective study to estimate the occurrence of A/CPA in a cohort of PA patients as a means to delineate a clinical strategy for intervening and caring for PA patients with autonomous cortisol secretion.

## 2. Materials and Methods

### 2.1. Study Subjects

We retrospectively reviewed the medical records of 555 consecutive patients diagnosed with PA between 2004 and 2015 in the endocrine department of our hospital (Chinese People's Liberation Army General Hospital). Among them, 414 were diagnosed with APA (414/555, 74.6%). Among these APA patients, 22 (22/414, 5.31%) had excess cortisol production. In addition, two patients each with unilateral adrenal nodular hyperplasia and bilateral adrenal adenomas presented with cortisol hypersecretion.

The diagnostic criteria of A/CPA were as follows: a confirmed PA diagnosis; confirmed glucocorticoid hypersecretion; absence of typical overt CS features, including easy bruising, striae, skin atrophy, facial plethora, and central obesity; presence of an adrenal mass confirmed via computed tomography before surgery; and a pathological diagnosis of the adrenal mass as an adrenal adenoma after surgery. The detailed diagnostic criteria for PA and APA are shown in Supplementary Data S1. All complications were diagnosed in the relevant departments of our hospital.

All 22 A/CPA patients and the other two patients had positive findings on the 1 mg overnight dexamethasone suppression test. Their serum cortisol levels were all above 1.8 *μ*g/dL (50 nmol/L) after the test.

We selected 80 contemporaneous pure APA specimens and six normal adrenal gland (NAG) tissue samples as controls in this study. The normal adrenal glands were obtained from renal carcinoma patients who were suspected of adrenal invasion and underwent radical nephrectomy and adrenalectomy. DNA from the tumor tissues of the 22 A/CPA patients were used for Sanger sequencing.

The present study was approved by the Protection of Human Subjects Committee of the Chinese People's Liberation Army General Hospital and written informed consent was obtained from all patients involved in this study.

### 2.2. RNA Isolation and Quantitative Real-Time PCR

We compared the mRNA levels of several steroidogenic enzymes necessary for both cortisol and aldosterone synthesis between A/CPA and pure APA tumor tissues by quantitative real-time PCR.

The relative mRNA levels of the 11*β*-hydroxylase (CYP11B1), aldosterone synthase (CYP11B2), and 17*α*-hydroxylase (CYP17A1) genes were standardized to GAPDH using the 2^−ΔΔCT^ method. The primer sequences are shown in Supplementary Table 1. Each sample was amplified in triplicate and the experiments were repeated three times.

### 2.3. Western Blot Assay

Pure APA and C/APA tissues were used in the western blot analyses against anti-CYP11B1, anti-CYP11B2, and anti-CYP17A1 antibodies. The antibodies were obtained from Santa Cruz (USA) and used at a dilution of 1 : 300. The mean densities of the bands were normalized to *β*-actin. The western blot experiments were performed as previously described [17].

### 2.4. Immunohistochemistry (IHC) Staining

Paraffin-embedded tumor tissue samples from A/CPA and pure APA patients were used for IHC staining analysis. The antibodies were the same as those used for the western blot experiments. The methods were performed as previously described [17].

### 2.5. DNA Extraction and Targeted Sequencing for Causative Mutations of Genes Involved in the Production of Aldosterone and Cortisol

Bidirectional Sanger sequencing was performed for targeted mutation detection in the KCNJ5, ATP1A1, ATP2B3, CACNA1D, CACNA1H, and PRKACA genes using DNA extracted from the tumor tissues of the 22 A/CPA patients. The DNA extraction and sequencing were performed as previously described [18]. The sequencing primers are shown in Supplementary Table 1.

### 2.6. Statistics

All quantitative data are shown as the means ± standard deviation. Comparisons between the A/CPA and pure APA groups were conducted by using Student's *t*-test or the *x* ^2^ test. Comparisons between the A/CPA, pure APA, and NAG groups were conducted by using analysis of variance. SPSS 18 software (SPSS Inc., Chicago, IL) was used in the data analysis. For all comparisons, *P* < 0.05 was considered statistically significant.

## 3. Results

### 3.1. Clinical and Hormonal Parameters in A/CPA and Pure APA Patients

The medical records of 22 A/CPA and 392 pure APA patients were assessed in this study. All patients underwent retroperitoneal laparoscopic adrenalectomy or partial adrenalectomy. Their clinical presentations are shown in Table 1. The age at onset was similar between A/CPA and pure APA patients (40.32 ± 6.37 versus 38.37 ± 7.76 years; *P* > 0.05). Women comprised 63.64% and 52.87% of the A/CPA and pure APA patients, respectively. The duration of hypertension before surgery was not significantly different between the two groups (11.91 ± 8.26 versus 9.46 ± 7.30 years; *P* > 0.05). In addition, there were no significant differences in body mass index or systolic and diastolic blood pressures (*P* > 0.05). The tumors were significantly larger in A/CPA patients than in pure APA patients (*P* < 0.05). On average, the largest tumor diameter in A/CPA patients was 6 mm longer than that in pure APA patients (24.50 ± 11.34 versus 18.92 ± 7.98 mm).

The proportion of patients with cardio-cerebrovascular events in A/CPA patients was significantly larger than that in pure APA patients (50% versus 16.07%; *P* < 0.0001). Strokes occurred in 36.36% of A/CPA patients and 10.46% of pure APA patients, but the difference was not statistically significant. For heart disease (cardiac complications), including atrial fibrillation, myocardial infarction, heart failure, and coronary atherosclerosis, the incidences differed significantly between the two groups (*P* < 0.001). In addition, the incidences of glucose intolerance/type 2 diabetes, osteopenia/osteoporosis, and metabolic syndrome differed significantly between the two groups (*P* < 0.05). However, there was no significant difference in the incidence of lipid metabolic disorder between the two groups (*P* > 0.05).

Each PA patient with an adrenal mass underwent a baseline cortisol secretion test (0:00, 8:00, 16:00 serum cortisol), and 63.77% underwent cortisol confirmation tests (1 mg overnight dexamethasone suppression test) for suspected cortisol dysrhythmia. The detailed methods are shown in Supplementary Data S1.

There were no significant differences in the baseline biochemical and hormonal parameters between A/CPA and pure APA patients, including hypokalemia, the aldosterone/renin ratio, and the serum potassium, plasma aldosterone, and serum aldosterone levels after saline infusion tests. The cortisol value after the 1 mg dexamethasone suppression test was significantly lower in the pure APA group than in the A/CPA group (*P* < 0.001). The detailed results are shown in Table 2.

### 3.2. Postsurgery Results

All A/CPA and pure APA patients benefited from surgery, with improved hypertension and hypokalemia after adrenalectomy. The preoperative management involved administration of 120 mg spironolactone daily for one week, glucose control, blood pressure control, and potassium correction. During and after the surgery, 100 mg hydrocortisone was injected intravenously and oral prednisone was administered as glucocorticoid replacement therapy and gradually reduced over 1 week until completely eliminated.

None of the A/CPA patients experienced postoperative adrenal crisis or adrenal insufficiency.

The postsurgery results are shown in Table 3. After surgery, 100% of the instances of hypokalemia disappeared and the blood pressure values returned to normal in 88.51% of pure APA and 100% of A/CPA patients without the use of antihypertensive medications. Postsurgery, their blood pressures reached the greatest drop within one month. The median time to return to stable normal blood pressures in both A/CPA and pure APA patients was one month. In the small number (11.49%) of pure APA patients with postoperative hypertension, their blood pressure was better controlled than before surgery with the use of a single antihypertensive agent. In addition, all abnormal hormone parameters returned to normal levels. No patients showed evidence of tumor recurrence by photography scanning.

### 3.3. Molecular Biological Studies of A/CPA and Pure APA Tissues

#### 3.3.1. Quantitative Real-Time PCR and Western Blot Analyses of CYP17A1, CYP11B1, and CYP11B2 mRNA Expression Levels

**Figure 1:**
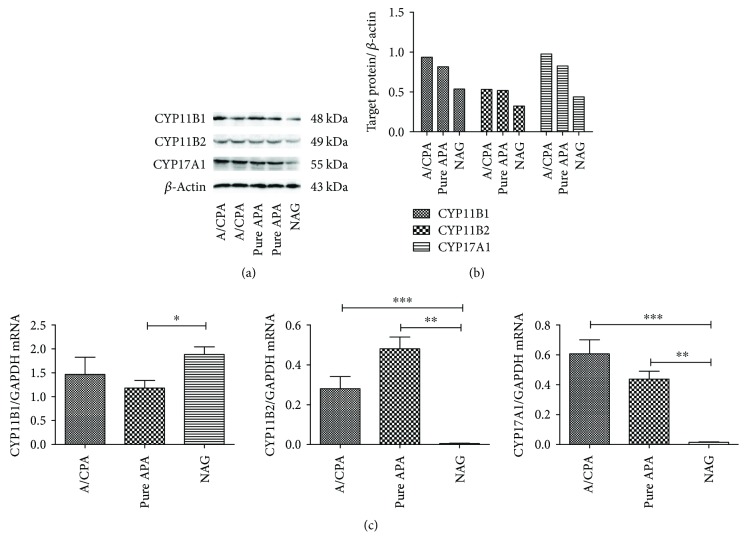
CYP11B1, CYP11B2, and CYP17A1 mRNA and protein expression levels in A/CPA tissue, pure APA tissue, and normal adrenal glands (NAGs). (a) Western blot showing CYP11B1, CYP11B2, and CYP17A1 protein bands in the A/CPA, pure APA, and NAG tissues. (b) The intensity of the CYP11B1, CYP11B2, and CYP17A1 protein bands compared with *β*-actin. (c) CYP11B1, CYP11B2, and CYP17A1 mRNA expressions in A/CPA, pure APA, and NAG tissues. ^∗^ *P* < 0.05, ^∗∗^ *P* < 0.01, ^∗∗∗^ *P* < 0.001.

The differences in the CYP17A1, CYP11B1, and CYP11B2 mRNA expression levels between the A/CPA and pure APA groups were not statistically significant (*P* > 0.05). However, the CYP17A1 mRNA expression levels were significantly higher in both the A/CPA and pure APA groups compared to those in the NAG group (*P* = 0.0004; *P* = 0.0072), as were the CYP11B2 expression levels (*P* < 0.001 for both). In the western blot analysis, CYP17A1, CYP11B1, and CYP11B2 bands were detected in both the A/CPA and pure APA tissues, with no significant differences between the groups (Figure 1).

#### 3.3.2. IHC Analysis

**Figure 2:**
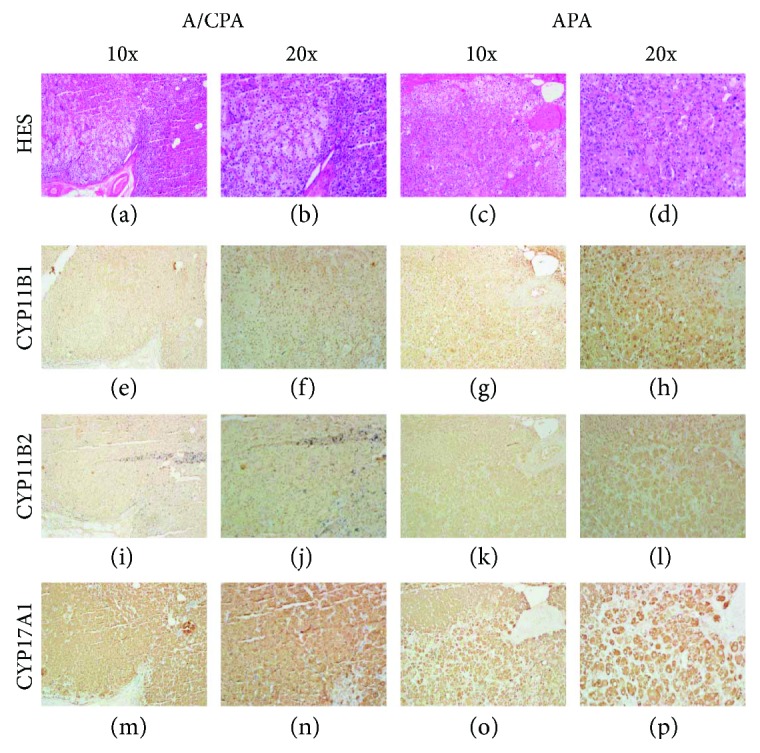
Immunohistochemistry results for CYP11B1, CYP11B2, CYP17A1, and hematoxylin-eosin stain (HES) in A/CPA and pure APA tissue.

Macroscopically, the adrenal adenomas showed a homogeneous golden yellow cut surface. In our IHC findings, both the A/CPA and pure APA tissues were positive for CYP17A1, CYP11B1, and CYP11B2. The pure APA tissue showed more intensive CYP11B1 and CYP11B2 staining compared to the A/CPA tissue. There was no visible difference in the staining intensity of CYP17A1 between the two groups, but a higher proportion of immune-reactive cells was identified in the A/CPA tissue compared to in the pure APA tissue. Histological examination revealed that the adrenal APAs exhibited large clear cells and small compact cells. Moreover, both types of cells were positive for CYP17A1 and CYP11B1, while CYP11B2 was mainly observed in the small compact cells. Upon hematoxylin-eosin staining, we observed that, in the A/CPA tumor tissue, the proportion of large clear cells was approximately 20% larger than that in the pure APA tumor tissue, accounting for 50% of cells. Conversely, in the pure APA tissue, the small compact cell proportion was beyond 60%. Moreover, KCNJ5-mutated tumor tissue showed a higher number of large clear cells than nonmutant tumor tissue. The IHC staining analysis results are shown in Figure 2.

#### 3.3.3. Target Sequencing for Causative Mutations of Genes Involved in the Production of Aldosterone and Cortisol

Seventeen DNA samples from the 22 A/CPA patients were found to harbor a KCNJ5 mutation. Both hotspot mutations (G151R and L168R) were detected, including two L168R mutations and 15 G151R mutations. However, no PRKACA hotspot mutations (L206R or C200_G201 ins V) were detected in these patient samples. Further, no ATP1A1, ATP2B3, CACNA1D, or CACNA1H mutations were detected.

In addition, we selected two prominent nodes from the unilateral adrenal nodular hyperplasia patient for Sanger sequencing. Both contained G151R mutations in the KCNJ5 gene, but no PRKACA mutations were observed. The patient with bilateral adrenal adenomas underwent partial adrenalectomy; her two adenomas were both without PRKACA mutations. However, the left one had a KCNJ5 mutation (G151R), while the right one did not have any KCNJ5 mutation.

## 4. Discussion

Among our 555 PA patients, 414 (74.6%) were identified as APAs, which is a higher proportion than in European and South Africa studies [19–21]. The prevalence of APA among PA patients at our institute is in accordance with that in Japanese PA patients (74% and 84% in two studies) [22, 23]. In distinct population groups, differences in genetic and lifestyle factors may affect the prevalence and presentation of APAs [24]. Further, the PA patients in our study may not represent the general PA population. Some APA patients with severe symptoms treated in other hospitals came to our institute for further diagnosis and operative treatment. These factors may influence the proportion of APA cases among PA patients in our hospital.

We identified 22 patients with aldosterone- and cortisol-cosecreting adrenal adenoma; the A/CPA incidence was 5.31% among APA patients at our institute, which was lower than the reported 21% in Japan from 38 PA patients [9] and 12.1% reported by Piaditis et al. [8]. Subclinical CS is characterized by subtle cortisol hypersecretion from the adrenal mass, without typical overt CS presentation. Previous studies have reported a prevalence ranging from 5–19% [25–28]. These data suggest that subtle cortisol hypersecreting adrenal tumors are not rare and may be underestimated due to the infrequent use of cortisol excess screening tests for nonfunctional adrenal tumors or APA patients.

In our findings, the tumors in A/CPA patients were approximately 6 mm larger than those of the pure APA patients, a finding compatible with data from previous A/CPA studies [9, 29]. APAs are composed of different cell types that may secrete cortisol [30]. A/CPA tumors may have a larger number and proportion of cortisol-producing cells compared to pure APA tumors; this observation may explain the larger A/CPA tumor size in this study. The A/CPA tumors were an average of 24.50 mm in diameter; histopathological examination confirmed that they were all adenomas. The other A/CPA cases from the publications also did not show any evidence of malignancy according to the Weiss criteria [31, 32].

The incidence of cardiovascular events in A/CPA patients was significantly higher than that of the pure APA patients (*P* < 0.0001). We evaluated the differences in the incidences of heart diseases, including atrial fibrillation, myocardial infarction, heart failure, coronary atherosclerosis, and stroke, between the two groups. There were 36.36% A/CPA patients and 10.46% pure APA patients with cardiac complications (*P* < 0.001). According to previous data, PA patients have a high risk of cardiovascular events, with a prevalence ranging from 11.3% to 35.2% [6, 33, 34]. This difference in incidence may be due to different ethnicities of the study patients. In our study, the patients with A/CPA had a higher risk for heart disease compared to the patients with pure APA, likely due to autonomous cortisol secretion from the tumors. Nakajima et al. reported that autonomous cortisol secretion from adrenal adenomas may be an independent risk factor of cardiovascular events [11, 35]. Several studies have shown that cortisol can form a cortisol-mineralocorticoid-receptor complex that mimics the effects of mineralocorticoids, enhancing the negative effects of excess aldosterone secretion [36, 37]. Thus, excess cortisol may lead to cardiovascular damage and, consequently, the higher prevalence of cardiovascular events in A/CPA patients.

Among cerebrovascular complications, 13.64% and 5.61% of A/CPA and pure APA patients, respectively, experienced strokes. However, the difference in incidence was not statistically significant, possibly due to the small number of stroke patients in the A/CPA group (*n* = 3). The incidence of stroke in the current study was lower than that of heart disease, similar to the findings of other studies [6, 34]. Nevertheless, a Japanese study reported an 18% incidence of stroke in PA patients [11], which was higher than the rate observed in the current study, with a higher rate of stroke than of heart disease. However, few studies have reported the incidence of stroke in PA patients, especially among those with A/CPA, and more detailed data on the incidence of cerebrovascular events are required to evaluate the damage due to A/CPA tumors.

The incidences of glucose intolerance/diabetes, metabolic syndrome, and osteopenia/osteoporosis were also significantly higher in the A/CPA patients than in the pure APA patients in the current study (*P* < 0.05). Though these patients presented without any typical symptoms of CS, these data suggest that the subtle cortisol excess was not completely asymptomatic. Moreover, the presentation of A/CPA patients was similar to that in subclinical CS [38, 39]. The damage severity may have varied due to the extent and duration of the excess cortisol secretion. Misinterpretation of the results of adrenal venous sampling can occur when the subtle excess of cortisol secretion is not recognized in A/CPA patients. In turn, this may lead to false negative results and a low selectivity index [10, 40].

All PA patients with an adrenal mass undergo cortisol screening test at our institute. Fortunately, in this study, PA patients with hypercortisolism were identified before surgery; therefore, none of our patients experienced postoperative adrenal crises due to formal perioperative preparation for hypercortisolism. In our long follow-up period, all patients benefited from their adrenalectomy. This finding suggests that surgery is a highly effective treatment. Postoperative adrenal crisis or insufficiency in patients with A/CPA is not rare, owing to inadequate endocrine function testing [16, 29, 32, 41, 42]. Therefore, we suggest that endocrine function screening is important in patients with adrenal tumors. Screening for excess cortisol secretion is necessary in PA patients.

In our molecular biological findings, quantitative real-time PCR analysis showed no significant differences in the CYP11B1 (11*β*-hydroxylase) and CYP17A1 (17*α*-hydroxylase) mRNA expressions between resected A/CPA and pure APA specimens. Thus, the autonomous cortisol secretion in the A/CPA tumors resulted in a subtle excess, leading to subclinical hypercortisolism rather than overt CS. The CYP11B2 (aldosterone synthase) and CYP17A1 mRNA expressions were significantly higher in both the A/CPA and pure APA tumors than in the normal adrenal cortex, indicating that the excess aldosterone and cortisol did come from the adenomas in these patients. IHC staining and western blot analyses revealed that both the A/CPA and pure APA tumor tissues were positive for CYP11B1, CYP11B2, and CYP17A1, implying that these tumors can produce both aldosterone and cortisol. Furthermore, in the IHC analysis, the A/CPA tumor tissues showed a higher proportion of CYP17A1 immune-reactive cells and large clear cells. This may be part of the reason why the A/CPA tumor tissues were larger and produced a subtle excess of cortisol. Other researchers have also reported CYP11B1, CYP11B2, and CYP17A1 expressions in both A/CPA and APA tumors, indicating that they are not rare conditions and also confirming that these tumors produce aldosterone and cortisol simultaneously [14, 32, 43–45].

Somatic mutations were recently identified in aldosterone- and cortisol-producing adenomas (APAs and CPAs, resp.). For APAs, mutations in the potassium inwardly rectifying channel, subfamily J, member 5 (KCNJ5) [46–48]; L-type and T-type voltage-gated calcium channels (CACNA1D, CACNA1H) [49–51]; and the P-type ATPase gene family (ATP1A1 and ATP2B) [51, 52] have been shown to be involved in extracellular and intracellular ion homeostasis, membrane potential regulation, and steroidogenesis and have been identified to increase aldosterone secretion and reduce serum potassium levels. KCNJ5 mutations are considered the most frequent mutations in APAs. Similarly, for CPAs, some somatic mutations, such as in the catalytic subunit of the protein kinase A (PRKACA) gene, the stimulatory G-protein *α*-subunit (GNAS) gene, and the beta-catenin (CTNNB1) gene, have been reported to cause an excess secretion of cortisol. Among these, PRKACA mutations are considered the most frequent mutations [53, 54]. There is also evidence of PRKACA and KCNJ5 mutations in A/CPA patients [44, 55]. Herein, we found 17 samples harboring KCNJ5 mutations among the 22 A/CPA patients, but no PRKACA, CACNA1D, CACNA1H, ATP1A1, or ATP2B mutations were detected. In our previous study, in which we detected relevant mutations in Chinese APA and CPA patients, the prevalence of KCNJ5 mutations was 75.4%, and that of PRKACA mutations was 40.4%; other mutations accounted for very small proportions (less than 10%) [18, 56]. Considering the high rates of mutations of KCNJ5 and PRKACA in APA and CPA, respectively, our findings suggest that A/CPA may be more similar to APA than CPA, and that the subtle hypercortisolism may result in a higher proportion and number of cortisol-producing cells due to reasons other than prominent causative mutations. Recently, a study investigated cortisol excess in patients with PA and found that the cosecretion of cortisol was independent of the primary aldosteronism subtype or tumor tissue genotypes. The authors also confirmed that cortisol excess, rather than mineralocorticoid excess, was closely linked to adverse metabolic risk in PA [57].

## 5. Conclusion

A/CPA patients have a high incidence of cardiovascular events and metabolic disorders. Screening for excess cortisol secretion is necessary for PA patients. We recommend general endocrine function screening in patients with adrenal tumors.

## Figures and Tables

**Table 1:** Clinical data of A/CPA patients and pure APA patients.

	A/CPA patients	Pure APA patients	*P* value
Number of cases	22	392	
Age (years)	40.32 ± 6.37	38.37 ± 7.76	**0.1255**
Age range (years)	26–51	19–58	
Female, *n* (%)	14, 63.64%	219, 52.87%	**0.3252**
BMI (kg/m^2^)	25.45 ± 3.22	25.28 ± 3.44	**0.8111**
Tumor size (mm)	24.50 ± 11.34	18.92 ± 7.98	**0.0318** ^∗^
SBP (mmHg)	181.4 ± 31.55	180.6 ± 26.57	**0.8180**
DBP (mmHg)	108.2 ± 13.26	109.2 ± 14.87	**0.7071**
Duration of HT (years)	11.91 ± 8.26	9.46 ± 7.30	**0.1489**
Stroke, *n* (%)	3 (13.64%)	22 (5.61%)	**0.1242**
Heart disease, *n* (%)	8 (36.36%)	41 (10.46%)	**0.0003** ^†^
CVE history, *n* (%)	11 (50%)	63 (16.07%)	**<0.0001** ^†^
Glucose intolerance/diabetes, *n* (%)	13 (59.09%)	85 (21.68%)	**<0.0001** ^†^
Lipid metabolic disorder, *n* (%)	9 (40.91%)	108 (27.55%)	**0.1757**
Osteopenia/osteoporosis, *n* (%)	3 (13.64%)	4 (1.02%)	**<0.0001** ^†^
Metabolic syndrome, *n* (%)	8 (36.36%)	72 (18.37%)	**0.0375** ^∗^
Cortisol excess screening, *n* (%)^‡^	22 (100%)	392 (100%)	—
Confirmation test performed, *n* (%)^§^	22 (100%)	264 (67.35%)	—
Aldosterone excess screening, *n* (%)	22 (100%)	392 (100%)	—
Confirmation test performed, *n* (%)	22 (100%)	392 (100%)	—

BMI: body mass index; SBP: systolic blood pressure; DBP: diastolic blood pressure; HT: hypertension; CVE: cardiovascular events; A/CPA: aldosterone- and cortisol-coproducing adenoma; APA: aldosterone-producing adenoma; ^∗^ *P* < 0.05; ^†^ *P* < 0.001; ^‡^Cortisol excess screening: 0:00, 8:00, 16:00 serum cortisol tests; ^§^Cortisol excess confirmation tests: 24-hour urinary free cortisol test and 1 mg overnight dexamethasone suppression test.

**Table 2:** Baseline biochemical and hormonal parameters of A/CPA and pure APA patients.

	A/CPA patients	Pure APA patients	*P* value
Hypokalemia (%)	90.91%	89.66%	1.000
Serum potassium (mmol/L)	2.537 ± 0.154	2.475 ± 0.560	0.9683
Plasma aldosterone (ng/dL)	23.49 ± 14.20	24.23 ± 6.986	0.2213
ARR	151.6 ± 146.3	139.1 ± 112.3	0.9664
Saline infusion test: aldosterone (ng/dL)	16.079 ± 4.310	21.845 ± 6.773	0.1397
Basal cortisol 8:00 am (nmol/L)	391.5 ± 135.6	343.6 ± 131.8	0.2033
ACTH 8:00 am (pmol/L)	5.974 ± 4.695	5.015 ± 2.781	0.9222
UFC/24 h (nmol/day)	455.6 ± 288.5	302.4 ± 204.7	0.1858
Cortisol after 1 mg dexamethasone test^∗^ (nmol/L)	119.5 ± 76.26	37.05 ± 10.024	0.0098^∗∗^
Cortisol after low-dose dexamethasone (nmol/L)	235.8 ± 234.3	—	—
Cortisol after high-dose dexamethasone (nmol/L)	116.5 ± 24.47	—	—

ARR: aldosterone-to-plasma renin activity ratio; ACTH: adrenocorticotropic hormone; UFC: urinary free cortisol; ^∗^the lowest cortisol value that can be detected in our laboratory is 25.7 nmol/L. Thus, the results of a fraction of pure APA patients who underwent the 1 mg dexamethasone test could not be used in this analysis due do cortisol levels < 25.7 mmol/L. ^∗∗^ *P* < 0.01.

**Table 3:** Postsurgical clinical and hormone parameters of A/CPA and pure APA patients.

	A/CPA patients	Pure APA patients
Normal serum potassium (%)	100%	**100%**
Serum potassium (mmol/L)	4.13 (3.54, 5.02)	**4.00** (**3.34**, **4.84**)
Normal BP without medication (%)	100%	**88.51%**
Systolic blood pressure (mmHg)	120 (110, 130)	**122.5** (**100**, **170**)
Diastolic blood pressure (mmHg)	80 (70, 90)	**80** (**70**, **110**)
Time to return to normal BP (months)	1 (0.25–8)	**1** (**0.25–10**)
Normal cortisol (%)	100%	**100%**
Basal cortisol 8:00 am (nmol/L)	424.00 (326.53, 563.60)	**410.60** (**352.77**, **453.03**)
Normal aldosterone (%)	100%	**100%**
Plasma aldosterone (ng/dL)	11.20 (7.27, 13.43)	**10.95** (**6.99**, **14.02**)
Tumor recurrence (%)	0	**0**

Unless otherwise specified, data are shown as the median and ranges.

## Supplementary Materials

Supplementary Materials Supplementary Data S1: the primary aldosteronism diagnostic criteria used at our institute. Supplementary Table 1: primer sequence.
