# Supplementary material for: Clinical Characteristics of Aldosterone- and Cortisol-Coproducing Adrenal Adenoma in Primary Aldosteronism
Source: Int J Endocrinol. 2018 Mar 25;2018:4920841. doi: 10.1155/2018/4920841 (PMC5889857; doi:10.1155/2018/4920841)
Supplement: Supplementary Materials — Supplementary Data S1: the primary aldosteronism diagnostic criteria used at our institute. Supplementary Table 1: primer sequence. [file 4920841.f1.docx]

**Supplementary data**

Primary aldosteronism in our institute was diagnosed as the following criteria:

**Screening test:**

1. **Hypertension (SBP/DBP >140/90 mmHg).** Blood pressure was checked after the patients rested for 30 minutes.

2. **Increased plasma aldosterone concentration (PAC >17.4ng/dL).**

3. **Aldosterone/ renin ration (ARR) >40ng/dL / ng/(mlh).** All the patients were stoped interfering antihypertensives and switched to little interfering medications for at least 2 weeks. while for spironolactone, at least 6 weeks before the evaluation. Patients should intake sodium >200 mmol (6 g) for 3 days and substantiated the 24-h urine sodium collection >200 mmol. Oral potassium chlorate was taking to maintain potassium within normal range.

At the time of blood sampling, patients were in the upright position for 2 hours after they woke up in the morning (8:00 am) and then in the seat for 10 minutes. Then we collected their blood to measure the plasm aldosterone concentration (PAC) and plasma renin activity (PRA).

Confirmatory test was performed on patients who were screened positive for PA.

**Confirmatory test:**

**Captopril loading test.** Patients were administrated 50 mg captopril after remaining standing at least 1h. Blood samples were taken before and 1 and 2h post captopril administration. If PAC was failed to suppress below 416 pmol/L (15 ng/dL), the primary aldosteronism was confirmed.

Or **Intravenous saline loading test**. The test was performed on patients after an overnight fast. 2L 0.9% saline was infused introvenously over 4 hours from 8:00 am to 12:00am. The patients were in bed throughout the infusion, the blood was taken at completion from the contralateral arm for PAC measuring. If PAC was failed to suppress below 277 pmol/L (10 ng/dL), the primary aldosteronism was confirmed.

**Adrenal computed tomography**

The contrast-enhanced CT scanning was performed on all patients with PA. Patients with bilateral lesions or normal-appearing adrenal glands on CT images were offered to adrenal venous sampling(AVS) for differentiating PH subtypes.

**Adrenal venous sampling** (AVS)

For AVS, we followed the same criteria as previously described. The placement of adrenal vein catheter was considered correctly if the cortisol gradient of adrenal vein/inferior vena cava was at least 2 and lateralization was determined when the aldosterone/cortisol ratio from one side was at least 4 times the ratio from the other side.

For each patient with adrenal masses or nodules underwent PA and Cushing’s syndrome screening test.

The Cushing’s syndrome initial testing was as following criteria:

Exclude exogenous glucocorticoid exposure

**Test of 0:00, 8:00,16:00 serum cortisol.** If the value and rhythm was disorder, the patient underwent 24-hour urine free cortisol test and 1-mg overnight dexamethasone suppression test

**Urine free cortisol test (24-UFC, two measurements).** The cut-off value was 5 times over the normal range.

**1-mg overnight dexamethasone suppression test (1-mg LDDST):** One milligram of dexamethasone was orally administered at 23:00, and blood for determination of cortisol was withdrawn at 09:00 the next morning. The cut-off value was 1.8 µg/dL (50 nmol/L).

**Supplementary Table 1:** Primer sequence

| Primer name | Primer sequence |
| --- | --- |
| **PRKACA1-F** | ATGACCACTGACGTTCACCT |
| **PRKACA1-R** | GGCTGGTCTGCGAAGAAG |
| **PRKACA2-F** | CGTCCTGACCTTTGAGTATCTG |
| **PRKACA2-R** | AGTCCACGGCCTTGTTGTA |
| **KCNJ5-E21F** | GATGGTGTCTTTTTAACTCAAAGC |
| **KCNJ5-E21R** | GTGATGACTCGGAAGCCATAC |
| **KCNJ5-E22F** | CTTTCCTGTTCTCCATTGAGACC |
| **KCNJ5-E22R** | CTGAGGAGGACAAAGCGCC |
| **KCNJ5-E3F** | ATGCATGTAACTTCCGTTTCCC |
| **KCNJ5-E3R** | GCCAGTGACAGGAGGTCTTAG |
| **ATP1A1-1F** | ATTATTCATGGAGGAATTTGCTAG |
| **ATP1A1-1R** | AATCCATATGCTGAATTACAGAAC |
| **ATP1A1-2F** | TTAGTCATCCTATGTAATTGTGTAAA |
| **ATP1A1-2R** | AGCGGAAGAGTGTAACATTC |
| **ATP1A1-3F** | TGACTCTATCGTTCATAAATGTTAA |
| **ATP1A1-3R** | CTAAGAGATGAAGCCAAGGA |
| **ATP1A1-4F** | ATGAAGTAAGTAATGAAGGACATG |
| **ATP1A1-4R** | TAGCTGCTATCATGGAAGC |
| **ATP2B3-1F** | CGTGTCCATACCTCTTCTTC |
| **ATP2B3-1R** | ACATTGTGCAGATGCTGAA |
| **ATP2B3-2F** | AGAGTGGTTTCAGACACAG |
| **ATP2B3-2R** | ATTCAGGACACAAAGCACT |
| **CACNA1D-1F** | TAGGAGCACTAACCTTCAG |
| **CACNA1D-1R** | ACGGAATCTCACAGACAGA |
| **CACNA1D-2F** | AGCTATAGATACGTAGATGTTTGG |
| **CACNA1D-2R** | AACCATGATC CACAAAGCA |
| **CACNA1D-3F** | TCTATCTCACATCCAGGCTT |
| **CACNA1D-3R** | TCAGTAAATGTGCTGGTATATTG |
| **CACNA1D-4F** | ACATGCCAACAGTGTATTCATA |
| **CACNA1D-4R** | CCTTACATAAACCATTCAGCC |
| **CACNA1D-5F** | ACACAGTAGAATACGTGGACA |
| **CACNA1D-5R** | TCTCTCCAGCATTCCATGT |
| **CACNA1D-6F** | ACTGTGAAAGGCAGCTTAA |
| **CACNA1D-6R** | AACTACTACCACCACCATC |
| **CACNA1D-7F** | AACCCACTCCTATGAGACCA |
| **CACNA1D-7R** | AAAGCATCTCAGAGGACACATA |
| **CACNA1D-8F** | GTCCTGCATGGGTGTTCTGA |
| **CACNA1D-8R** | ACGAAGTGCTTTTCGGGGAA |
| **CACNA1D-9F** | CACGCTAACTGTGCAGGGA |
| **CACNA1D-9R** | TCAGCTCTGCCCAGAAGAG |
| **CACNA1D-10F** | CCAATCTACAACCACCGCGT |
| **CACNA1D-10R** | GACCAAGGGACAGAAGCCAA |
| **CACNA1D-11F** | ACGGTTCTTCCTCACTGTCG |
| **CACNA1D-11R** | CTTCAGCAGAGGCATTTGGCT |
| **CACNA1H-F** | ACCCCTGGAT GCTGCTGTAC |
| **CACNA1H-R** | GTGCTGCCGGCACTTGTGAAG |
